# Congenital Teeth

**Published:** 1897-04

**Authors:** E. A. Benton

**Affiliations:** Central City, Neb.


### Congenital Teeth.

BY E. A. BENTON, M. D., CENTRAL CITY, NEB.

On reading Dr. McKee's article in the *Journal of the American Medical Association*, page 253, February 6, 1897, I was so impressed with its statements in regard to the etiology, treatment and non-frequency of the cases of congenital teeth, that it seemed to be my duty to register my personal experience in these cases. Dr. McKee says, "this freak of nature has been noticed at wide intervals and with great rarity for a long time." Also "these cases serve as a curiosity for doctors and students, and are a sight many do not see in a lifetime." I had not supposed these cases so rare or so much of a curiosity, for, in a practice of thirty years, with a fair obstetrical clientage, I have attended three cases where there were two lower central incisor teeth at birth; and I never failed at these cases, or soon after, while it was the talk of the neighborhood, to find several old ladies who had *seen* or *heard* of several such cases. My wife, who is now 50 years old (don't let her know that I am telling her age, for she looks to be only 40), informs me that she had two central inferior incisors at birth, and that they remained the usual length of time, when they were replaced by permanent teeth, which she still has in a perfect state of preservation. Her family and personal history is perfect. My first case was at my wife's third confinement, resulting in twins, male and female; born at full term, both strong and healthy, with nothing out of the usual line, except the girl had two central inferior incisor teeth, firmly set in gums and well developed, but a little smaller than ordinary milk-teeth developed at the natural time. The teeth were in healthy condition when she died of cholera infantum at the age of four months. My second case was at my wife's fifth and last confinement, when a strong and healthy daughter was born with two central inferior incisor teeth; one firm in the gums, and the other loose and attached only to the outer gum. I removed it with my fingers without producing any hemorrhage or soreness. It has never been replaced. The other is firm and in a good state of preservation, but smaller than to correspond with the other teeth. She is seven years old, and a well-developed, strong, healthy child. I am somewhat anxious to see if a permanent tooth will be developed where the one was extracted. My third case was a fine, healthy boy, fifth child of Mr. and Mrs. J. E. B., both parents healthy, and with good family history. This child was born at full term, with same teeth as other cases, being as large as ordinary milk-teeth and firmly set in gums. At the age of three weeks the teeth had made an intractable ulcer under the child's tongue, rendering nursing impossible. I extracted them. No hemorrhage or other trouble followed. The family soon moved from the city, and I have lost track of them. My cases would indicate that no milk-teeth would come to fill the vacancy caused by the exfoliation or extraction of congenital teeth; also that rickets or improper nutrition was not a causative factor in their development. It seems to me to be a more rational conclusion to assign the appearance of congenital teeth to a precocious or premature occurrence of the normal processes in the development and cutting of the milk-teeth. We certainly have this precocity in the development and physiologic action of other parts of the human economy.

---

A BLISTER applied over the thickened tissues around a callous ulcer is one of the most rapid methods by which the disappearance of the exuded materials can be brought about.—*W. W. Cheyne.*

